# Resting-State Brain Dynamics Unique to Anxiety in Major Depressive Disorder

**DOI:** 10.1155/2024/4636291

**Published:** 2024-07-13

**Authors:** Yingying Du, Qianyi Luo, Yurong Zou, Huiqin Nie, Yuhong Li, Xiaohui Lin, Herui Shang, Hongjun Peng

**Affiliations:** ^1^Department of Clinical Psychology, The Affiliated Brain Hospital of Guangzhou Medical University, Guangzhou 510370, China; ^2^Guangdong Engineering Technology Research Center for Translational Medicine of Mental Disorders, Guangzhou 510370, China; ^3^Department of Publicity and Health Education, Shenzhen Longhua District Central Hospital, Shenzhen 518000, China; ^4^Department of Health Management, Guangzhou Medical University, Guangzhou 511436, China

## Abstract

**Background:**

Major depressive disorder with anxiety (MDD-A) is considered as a clinical subphenotype of major depressive disorder (MDD). There continues to be debate regarding the legitimacy of differentiating between the two diagnoses and their neurobiological foundations, given that the symptoms of MDD and MDD-A overlap. However, there is still a dearth of research that delineates the dynamic alteration in the brain activity unique to anxiety in MDD with resting-state functional magnetic resonance imaging (R-fMRI).

**Methods:**

30 patients with MDD, 45 patients with MDD-A, and 46 healthy controls completed R-fMRI scans. Dynamic analysis was utilized to generate many widely used measures, such as voxel-mirrored homotopic connectivity, global signal correlation, regional homogeneity, amplitude of low-frequency fluctuations, and network degree centrality. Concordance between these indices was assessed with Kendall's W coefficient for both volume and voxel-wise concordance. Finally, the differences in voxel-wise concordance among the groups were looked at, and their relationship to clinical factors was assessed.

**Results:**

Compared to the healthy control group, both MDD and MDD-A exhibited decreased dynamic R-fMRI indices in the bilateral calcarine, left postcentral gyrus, inferior parietal lobe, right lingual gyrus, and middle occipital gyrus. In comparison to the MDD group, the MDD-A group displayed a reduction in voxel-wise concordance in the left medial superior frontal gyrus. Furthermore, it was observed that the MDD and MDD-A groups both exhibited a negative correlation between anxiety levels and voxel-wise concordance in the left medial superior frontal gyrus.

**Conclusions:**

The aberrant voxel-wise concordance of the left medial superior frontal gyrus may differentiate the neurobiological aspects of MDD with anxiety symptom from MDD. These findings indicate the underlying mechanisms implicated in MDD with anxiety symptom while highlighting the significance of accounting for heterogeneity in depression research.

## 1. Introduction

Major depressive disorder (MDD) as a major cause for global morbidity and mortality imposes a significant economic burden on public health, especially for developing countries [1–3]. The cooccurrence of MDD and other psychiatric disorders, particularly anxiety disorders, is highly prevalent; about 40-70% of patients with a current diagnosis of depression have anxiety symptoms or disorders. [4, 5]. Patients with MDD who have anxiety symptoms report a greater degree of cognitive impairment [6], poorer response to treatment [7, 8], and a heightened risk of suicidal behaviors [9]. In addition to coexisting, depression and anxiety symptoms can alternate and transform, making diagnosis and treatment complicated [10, 11]. There is still a limited understanding of the neurobiology underlying anxiety symptoms in patients with MDD.

Neuroimaging studies using static analyses of R-fMRI data have previously shown abnormalities in limbic, prefrontal, and subcortical regions in MDD patients [12, 13], whereas MDD patients with coexisting anxiety symptoms or disorders showed altered brain regions including the anterior cingulate cortex, amygdala, hippocampus, and orbital frontal cortex [14, 15]. However, converging evidence indicates that intrinsic brain activity is changing from time to time, and these dynamic characteristics can be captured by the sliding window method of dynamic analysis [16–18]. Compared to the conventional analysis of R-fMRI data, the dynamic analysis interrogates changes more precisely in intrinsic brain activity across time periods by calculating R-fMRI indices at seconds' scale, which can be helpful in attempts to understand different aspects of MDD. For instance, Xue et al. [19] conducted a study on gene expression profiles linked to altered dynamic regional homogeneity (dReHo) in MDD. Using dynamic amplitude of low-frequency fluctuations (dALFF), Nie et al. [20] have further insights into the antidepressant effects of electroconvulsive therapy. Additionally, Li et al. [21] have employed dynamic voxel-mirrored homotopic connectivity (dVMHC) to characterize abnormalities in brain regions in MDD with suicidal ideation. When exploring the functional integration of the intrinsic brain, Sheng et al. [22] conducted dynamic network degree centrality (dDC) to identify typical features of brain functional integration dysfunction in patients with MDD. Dynamic global signal correlation (dGScorr) is then utilized to examine the correlation between altered global and local neural activity, providing insights into the physiological mechanisms of balance dysregulation between brain networks in MDD [23]. Accordingly, the present study makes use of the abovementioned temporal dynamic R-fMRI indices to offer novel understandings of the neurophysiological mechanisms of MDD patients with anxiety symptoms.

Currently, studies have made progress in examining the differences between MDD and anxious depression in response to antidepressant treatment, symptom severity, and cognitive functioning [24, 25]. Zhou et al. [26] found that anxiety symptoms can impact various physiological measures in patients with MDD, including blood pressure, thyrotropin levels, and levels of antithyroglobulin and thyroid peroxidase antibodies. Furthermore, postpartum depression with anxiety displays dynamic changes in functional connectivity between the subgenual anterior cingulate cortex and superior temporal gyrus compared to postpartum depression without anxiety [27]. The above evidence supports the idea that MDD with anxiety symptoms constitutes a separate clinical phenotype of MDD. Therefore, MDD with anxiety symptoms is suggested to have some common and unique neurobiological mechanisms compared to MDD. However, there is a paucity of literature that explores the relationship between neurobiology and underlying anxiety symptoms in patients with MDD.

In this investigation, the primary goal was to explore anomalous modifications in intrinsic brain activity between patients with MDD and MDD patients with anxiety symptoms (MDD-A), utilizing temporal dynamic analysis. We exposed brain functional couplings through volume and voxel-based concordance and gauged dynamic regional brain activity through R-fMRI measurements such as dALFF, dDC, dReHo, dVMHC, and dGSCorr. Consequently, we hypothesized that MDD-A exhibits distinct regional brain abnormalities in comparison to other groups. Secondly, these specific regional brain abnormalities are involved in the degree of anxiety.

## 2. Materials and Methods

### 2.1. Participants

A total of Seventy-five MDD patients were enlisted from the Guangzhou Medical University's Affiliated Brain Hospital in China. Forty-six individuals from the local community of Guangzhou were included as healthy controls. Depression diagnoses were determined by two clinicians at the Affiliated Brain Hospital of Guangzhou Medical University, China, using diagnostic criteria from the Diagnostic and Statistical Manual of Mental Disorders, Fifth Edition. To confirm the absence of psychiatric disorders, healthy controls underwent comprehensive screening. All excluded participants had (1) a past medical history of drug misuse or addiction, substance-induced mood disorders, bipolar illness, or schizophrenia; (2) a history of past or current anxiety disorders, including panic disorder, social phobia, general anxiety disorder, and agoraphobia; (3) a past medical history of any major physical or neurological condition; (4) a head injury with unconsciousness in the past; (5) pregnant or not suitable for an MRI; (6) left-handed.

MDD patients were categorized based on previous studies using the Hamilton rating scale for anxiety (HAMA) with a threshold score of 14 or higher [28]. Therefore, patients with MDD who scored 14 or higher on the HAMA scale were assigned to the MDD-A group, while those who scored less than 14 were assigned to the MDD alone group.

The research was authorized by the ethics council of the Guangzhou Medical University Affiliated Brain Hospital. All participants gave written permission after being fully informed about the research.

### 2.2. Measures

The 17-item Hamilton rating scale for depression (HAMD-17), consisting of 17 items, is a frequently utilised instrument for objectively evaluating the level of depression in MDD [29, 30].

The 14-item Hamilton rating scale for anxiety (HAMA-14) is an objective tool routinely employed in clinical settings to gauge and measure levels of anxiety [31]. A total score of ≥20, in accordance with the thresholds specified in the generalised anxiety disorder study, was considered an elevated level of anxiety condition [32, 33].

### 2.3. MRI Acquisition

Magnetic resonance imaging scans were conducted utilizing the 3 Tesla Philips MRI Scanner at the Radiology Department of the Brain Hospital, Guangzhou Medical University in China. All subjects were encouraged to close their eyes, relax, and remain motionless and alert. The echo planar imaging sequence parameters used for the resting-state functional scan were as follows: a repetition time of 2,000 ms, an echo time of 30 ms, 33 slices, a 90° flip angle, a 64 × 64 matrix, a 220 × 220 mm field of view, a 4 mm slice thickness, and a 0.6 mm inter-slice gap. The eight-minute scanning operation included 240 time points. As for the T1 sagittal images, their parameters are as follows: 188 slices with a thickness of 1 mm, a flip angle of 7 degrees, an acquisition matrix of 256 × 256, a repetition time of 8.2 ms, an echo time of 3.7 ms, and a voxel size of 1 mm × 1 mm × 1 mm.

### 2.4. Preprocessing of Magnetic Resonance Imaging Data

All preprocessing was carried out using the R-fMRI Processing Assistant (DPARSFA, http://www.restfmri.net/forum/DPARSF) and Brain Imaging Processing and Analysis software (DPABI2 version 4.5) [16]. To allow the signal to equilibrate and the subject to adjust to the scanning noise, we excluded the initial ten volumes of each participant. The remaining images have been adjusted for slice acquisition time delay. The Jenkinson model was then used to determine head motion by averaging the mean framewise displacement (FD) across all points in time of every participant [34]. The mean head movement data for all participants had a difference of only 0.2 mm among them. The individual structural pictures (T1-weighted MPRAGE) were then linearly transformed with 6 degrees of freedom to match them to the mean functional image [35]. The motion-corrected functional image space is standardized to the Montreal Neurological Institute space of 3 mm × 3 mm × 3 mm during the uniform segmentation process. While the remaining photos were filtered but not smoothed, the images used to compute ALFF and fALFF were smoothed without filtering. White matter signal and cerebrospinal fluid signal were viewed as confounding factors to be excluded by regression. The standardized pictures were subsequently subjected to a 4 mm full-width at half maximum Gaussian kernel to generate ALFF and fALFF [36]. Subsequently, ReHo, VMHC, and DC were calculated on standardized photos using Friston 24 head motion parameters [36]. Lastly, the images were filtered through a temporal bandpass between 0.01 and 0.08 Hz.

### 2.5. Computation of Multiple R-fMRI Indices

#### 2.5.1. ALFF and fALFF

ALFF and fALFF were utilised for the description of the regional features of an individual voxel, with a higher ALFF value indicating an increased level of brain activity in that region. The blood oxygen level signal is converted by Fourier decomposition from the time to the frequency domain and then squared. The average of the square root of the low amplitude range of 0.01 to 0.1 Hz is used to calculate the ALFF value [37]. fALFF is the ratio of the sum of the amplitudes of a given low frequency band to the sum of the Fourier amplitudes over the entire frequency range and is the normalized version of the fractional ALFF [38, 39].

#### 2.5.2. ReHo

ReHo is a measure of the degree of functional synchronization between a voxel and its neighbors. Higher ReHo values indicate greater connectivity and centrality of regional brain activity. This metric assesses the coherence between regions through calculating the Kendall coefficient between a given time sequence and those of its immediate neighbors [40].

#### 2.5.3. GSCorr

GSCorr is the Pearson correlation coefficient between the global mean series of all grey matter voxels and the time series of each voxel. These GSCorr values were then transformed using Fisher's *Z* method to obtain a normal distribution.

#### 2.5.4. VMHC

VMHC quantifies the functional connectivity between geometrically corresponding hemispheres. Pearson's correlation coefficients were computed between each time course and that of their contralateral counterparts. The above VMHC values were then subjected to Fisher's *Z* transformation to achieve normality of distribution [41].

#### 2.5.5. DC

DC was used to assess the connectivity of a given voxel to the brain to reflect its functional importance, using a graph-theoretic approach. The functional connectivity matrix of the whole grey matter was produced by computing Pearson's correlation coefficients on the time series of each paired voxel. DC was determined as the overall functional connectivity (defined as functional connectivity values above a 0.25 threshold) between a specific voxel and the remaining voxels [41, 42].

Our study examines the dynamic R-fMRI indices, comprising dALFF, dfALFF, dReHo, dGSCorr, dVMHC, and dDC, as an exploratory investigation to determine their dynamic and concordance differences between MDD, MDD-A, and healthy controls. According to this study, the significant differences in variability were observed for dALFF, dReHo, dGSCorr, and dDC. For the other indices, no significant variability difference was observed.

### 2.6. Dynamic R-fMRI Indices Calculation

This study employed the temporal dynamic analysis toolkit on DPABI2 (version 4.5) to compute dynamic indices [16]. The dynamic R-fMRI index changes throughout the brain were calculated using the sliding window method in this study. Window length is critical but flexible when calculating dynamic fMRI indexes. According to earlier research, the ideal window length to get accurate estimations of intrinsic brain activity while also recording quickly changing dynamic brain activity is 50 TRs [43, 44]. Therefore, this investigation implemented a window length of 50 TR alongside a step size TR of 1, yielding 181 windows over each participant.

The fALFF, ReHo, VMHC, DC, and GSCorr were calculated for each time window for each participant. To describe the dynamics of the indices, the mean and standard deviation (SD) maps were calculated for each index over the time window, and the SD of all windows was used to measure the dynamic. We conducted a one-way ANOVA analysis with post hoc Bonferroni correction to compare standardized SD maps among different groups. The SD maps were standardized to *z*-scores using *z*-transformation, and these standardized values were then used for statistical analysis.

### 2.7. Concordance Analysis

The combination of these indicators in the analysis can have a positive impact on their explanatory power, even though they have different meanings. Therefore, to determine the coupling of multiple different indices in different states, a concordance analysis was carried out to show their concordance quantitatively [16, 18]. Concordance indicates the level of integration in the brain, with higher levels of concordance leading to increased connectivity both within and between brain networks.

#### 2.7.1. Volume-Wise Concordance

The Kendall's W coefficient of five R-fMRI indices was computed for all voxels in each time window across the grey matter to determine spatial concordance values. The mean spatial concordance values for each participant throughout all time frames were computed to get the volume-wise concordance value.

#### 2.7.2. Voxel-Wise Concordance

The dynamic voxel-wise concordance indices were computed for each participant by calculating Kendall's W coefficient of five R-fMRI indices across all time windows. Then, the dynamic voxel-wise concordance indices were *Z*-normalized for subsequent analysis.

### 2.8. Statistical Analysis

Age, educational level, mean FD were compared between MDD, MDD-A, and healthy controls with one-way analysis of variance (ANOVA) and post hoc Bonferroni correction. Furthermore, we conducted a two-sample *t*-test to evaluate the comparison of clinical scale scores between the two populations, MDD and MDD-A. The statistical analyses were carried out using IBM Corp.'s SPSS software (version 19.0) based in Armonk, NY, USA.

In this research, a one-way ANOVA was utilised to compare standardized SD maps across various groups. Post hoc analysis was conducted using Bonferroni correction to account for multiple comparisons. When evaluating variances in the dynamic indices, the one-way ANOVA was carried out while using age, gender, education level, and mean FD as confounders. Additionally, a cluster size greater than 15 voxels and a significance criterion of *p* < 0.05 were used for the family-wise error correction (FWE) [45]. A significance level of *p* < 0.0125 was considered significant for regions with dALFF disparities due to multiple comparisons. This decision was made based on four significant clusters resulting from the dALFF analysis. For regions where variations in dDC were observed, significance was accepted at a level of *p* < 0.025 due to multiple comparisons. This decision was based on the identification of two significant clusters through the dDC analysis.

Differences in the volume-wise concordance between MDD, MDD-A, and healthy controls were compared using one-way ANOVA with Bonferroni post hoc correction while controlling for gender, age, educational level, and mean FD. The statistical analysis determined the significance level of difference for this study as a *p* value less than 0.05 with two tails.

Differences in voxel-wise concordance were evaluated between three groups employing a voxel-based one-way ANOVA with Bonferroni post hoc correction in SPM8 while controlling for gender, age, educational level, and mean FD. The mean dynamic index values of the brain regions that displayed statistically significant differences between groups in the voxel dynamic analyses were then extracted. For voxel-wise concordance analysis, *p* < 0.016 was considered significant in regions where differences were observed (analysis revealed three significant clusters). In the present investigation, the relationship between voxel-wise concordance and HAMA scores in individuals diagnosed with MDD was examined using (*p*_adj_ < 0.05 accepted as significant).

## 3. Results

### 3.1. Demographic and Clinical Characteristics

Table 1 presents the demographic and clinical information regarding the 75 participants diagnosed with MDD and 46 healthy controls. The MDD sample was divided into two groups, MDD-A (*n* = 45) and MDD (*n* = 30). Age, gender, and mean FD were not significantly different between the three groups. Additionally, both MDD and MDD-A groups showed no significant differences in HAMD scores. One-way ANOVA analysis shows significant differences in educational level among the three groups. The MDD-A group exhibited substantially higher HAMA than the MDD group.

### 3.2. Dynamic of R-fMRI Indices

**Figure 1:**
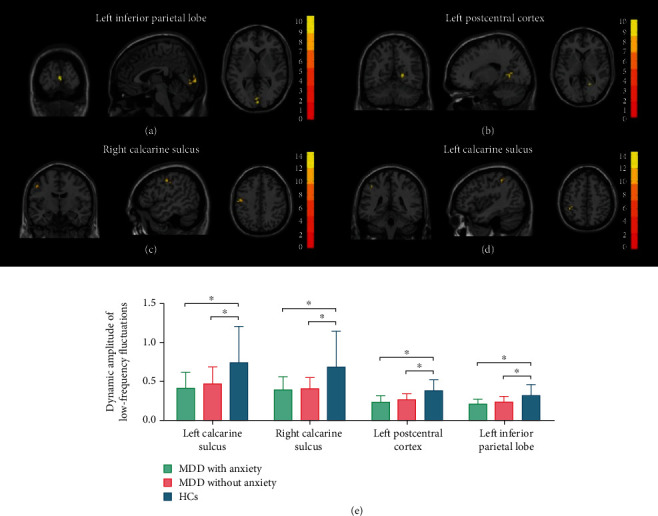
Regions with differences in dynamic amplitude of low-frequency fluctuations between the MDD with anxiety, MDD without anxiety, and HCs and post hoc analysis. (a, b) The intergroup difference in dynamic amplitude of low-frequency fluctuations in the left inferior parietal lobe and the left postcentral cortex. (c, d) The intergroup difference in dynamic amplitude of low-frequency fluctuations in the bilateral calcarine. (e) The multiple comparisons in regions with differences in dynamic amplitude of low-frequency fluctuations. MDD: major depressive disorder; HCs: healthy controls. ⁣^∗^The *p* value has reached a significant level. In the multiple comparisons in regions with differences in dynamic amplitude of low-frequency fluctuations, *p* < 0.05/4 = 0.0125 was accepted as significant.

**Figure 2:**
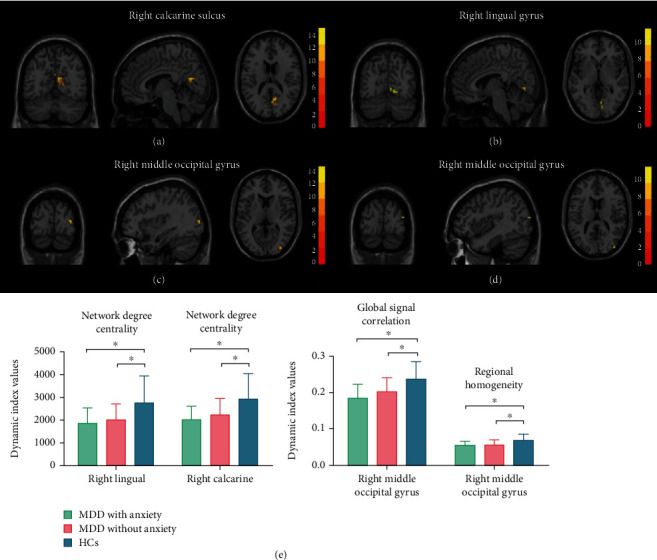
Regions with differences in dynamic network degree centrality, dynamic global signal correlation, and dynamic regional homogeneity between the MDD with anxiety, MDD without anxiety, and HC groups and post hoc analysis. (a, b) The intergroup difference in dynamic network degree centrality in the right calcarine sulcus and the right lingual gyrus. (c) The intergroup difference in dynamic global signal correlation in the right middle occipital gyrus. (d) The intergroup difference in dynamic regional homogeneity in the right middle occipital gyrus. (e) The multiple comparisons in regions with differences in dynamic network degree centrality, dynamic global signal correlation, and dynamic regional homogeneity, respectively. MDD: major depressive disorder; HCs: healthy controls. ⁣^∗^The *p* value has reached a significant level. In the multiple comparisons in regions with differences in dynamic network degree centrality, *p* < 0.05/2 = 0.025 was accepted as significant. In the multiple comparisons in regions with differences in dynamic global signal correlation and dynamic regional homogeneity, *p* < 0.05/1 = 0.05 was accepted as significant.

The ANOVA showed differences in dALFF, dDC, dGSCorr, and dReHo among the three groups (Table 2). The post hoc tests showed that both MDD and MDD-A had significantly lower dALFF in the bilateral calcarine sulcus, left postcentral gyrus, and left inferior parietal lobe than healthy controls (Table 3 and Figure 1). The dDC exhibited lower levels among patients with MDD and MDD-A in the right lingual gyrus and the right calcarine sulcus in comparison to healthy controls (Table 3 and Figure 2). In addition, MDD and MDD-A patients had significantly lower right middle occipital gyrus dGSCorr as well as dReHo than healthy controls (Table 3 and Figure 2).

### 3.3. Volume-Wise Concordance R-fMRI Indices

**Figure 3:**
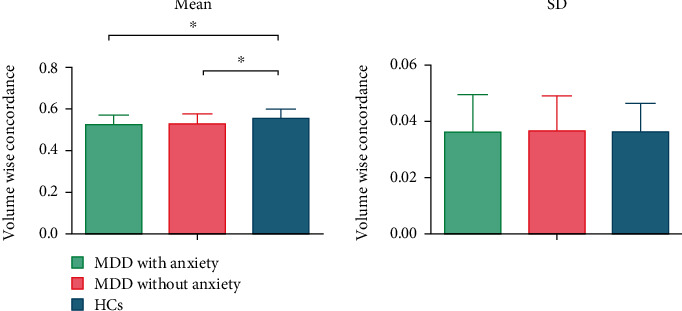
Comparison of volume-wise concordance among the MDD with anxiety, MDD without anxiety, and HCs. MDD: major depressive disorder; HC: healthy control. ⁣^∗^*p* < 0.05.

Mean volume-wise concordance significantly varies across healthy controls, MDD, and MDD-A (Table 4). The average concordance was found to be lesser in the MDD and MDD-A groups as compared to the HC group (Table 5 and Figure 3).

### 3.4. Voxel-Wise Concordance R-fMRI Indices

**Figure 4:**
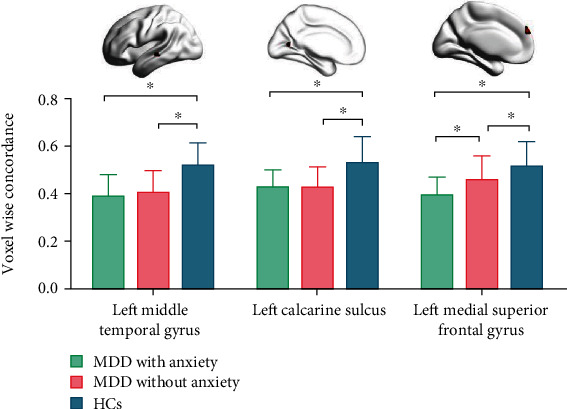
Regions with differences in the voxel-wise concordance of R-fMRI indices among the MDD with anxiety, MDD without anxiety, and HCs and post hoc analysis. MDD: major depressive disorder; HC: healthy control. ⁣^∗^The *p* value has reached a significant level. In the multiple comparisons in regions with differences in voxel-wise concordance, *p* < 0.05/3 = 0.016 was accepted as significant (voxel-wise concordance analysis resulting in three significant clusters).

**Figure 5:**
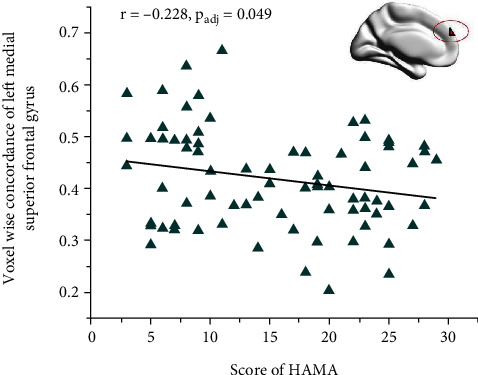
Correlation between score of HAMA and voxel-wise concordance. ⁣^∗^*p*_adj_ < 0.05 accepted as significant.

When voxel-wise concordance between HCs and MDD and MDD-A was compared, significant differences were found in the left medial superior frontal gyrus, left calcarine sulcus, and left middle temporal gyrus (Table 6 and Figure 4). Multiple comparisons demonstrated that MDD-A exhibited inferior concordance of the left medial superior frontal gyrus in comparison to both healthy controls and MDD (Table 7 and Figure 4). In addition, the HAMA score showed a negative correlation with a voxel-wise agreement in the left medial superior frontal gyrus (see Table 8 and Figure 5).

## 4. Discussions

Through the temporally dynamic analysis of R-fMRI, this research conducted a detailed investigation of the aberrant dynamics and concordance of intrinsic brain activity in patients diagnosed with MDD and MDD-A, revealed the abnormal dynamic features of MDD and MDD-A, and discovered the specific dynamic features of MDD-A. MDD and MDD-A exhibit significantly reduced dALFF in the bilateral calcarine, left postcentral gyrus, and inferior parietal lobe, as well as reduced dDC in the right lingual gyrus and right calcarine, when compared to the healthy control group. Additionally, decreased dGSCorr and dReHo were observed in the middle occipital gyrus in both MDD and MDD-A. Both MDD and MDD-A exhibited a common volume-wise decrease in total grey matter when compared to HCs, accompanied by a reduction in voxel-wise concordance in the left middle temporal gyrus, calcarine sulcus and medial superior frontal gyrus in MDD and MDD-A, which together quantify spatial and temporal decoupling. In addition, the MDD-A group showed reduced voxel-wise concordance in the left medial superior frontal gyrus compared to both the HCs and the MDD group, which was negatively correlated with the level of anxiety.

In this study, we observed a reduction dALFF in the bilateral calcarine sulcus, as well as a reduction a reduction dDC in the right calcarine sulcus and lingual gyrus in both MDD and MDD-A groups. The calcarine and the lingual gyrus are components of the primary visual cortex of the brain [46]. The right lingual gyrus plays a crucial role in visual memory [47] and is associated with processing words or overall shapes [48]. Research indicates that individuals with MDD exhibit anomalous neural activity in the calcarine sulcus, which is associated with impaired visual processing as well as dysfunction in the visual cortex [49–51]. Furthermore, a longitudinal investigation demonstrated that the functional steadiness of the calcarine sulcus during the baseline session predicted the amelioration of depressive symptoms in MDD patients [52]. Subsequently, positive correlations were found between ALFF values in the right lingual gyrus and anxiety scores in patients with migraine and anxiety comorbidity [53]. Additionally, both dynamic and static functional analyses have linked reduced functional connectivity of the calcarine and other brain regions with anxiety symptoms, affecting emotional processing and visual biases associated with fear [54, 55]. Using a temporal dynamics analysis, we provide evidence for abnormal temporal dynamics, suggesting that reduced activity in the calcarine sulcus may affect the modulation of symptoms of depression and anxiety.

The left inferior parietal lobe plays a central role in the dorsal attention network [56, 57], which is involved in the voluntary reorientation of attention and behavior in response to interoceptive stimuli and is highly activated in tasks that require the simultaneous processing of internal sensory/emotional information and cognitive demands [58, 59]. During cognitive tasks using R-fMRI, such as the parametric Go/No-Go test, the MDD comorbid anxiety group showed significantly less activity in the left inferior parietal lobe, suggesting that the level of the inferior parietal lobe activation could potentially discriminate between MDD comorbid anxiety patients and those with MDD alone [60, 61]. Further evidence that the parietal region is the brain area most associated with anxiety comes from a study using repetitive transcranial magnetic stimulation, which demonstrates that decreasing the electrical activity of the intraparietal sulcus when a sudden hazard reduces the physiological arousal associated with anxiety and fear [62]. According to Bishop et al. [63], neurocognitive model proposes that impaired cognitive control over threat-related information is a crucial factor in anxiety, and that anxious individuals selectively attend to threatening stimuli during the cognitive process of attentional orienting. He, then, suggested that the neural circuits that are characteristically dysfunctional in anxiety, that is, hyperresponsiveness of the amygdala and hypo recruitment of the prefrontal lobe, may be involved in the development of anxiety [64]. In summary, the abnormal process of orienting attention is specifically associated with anxiety and may serve to differentiate between depression alone and depression combined with anxiety disorder. In this study, a reduction in dALFF in the left parietal lobe was found in both MDD and MDD-A patients, suggesting the neurobiological changes in MDD with anxiety symptoms and MDD comorbid anxiety disorders are not equivalent. Therefore, it is inappropriate to consider the two disorders as identical.

The present results showed that MDD and MDD-A displayed a reduction in voxel-wise coherence within the left middle temporal gyrus, left calcarine sulcus, and left medial superior frontal gyrus compared to healthy controls. The findings indicate persistent yet uncoordinated intrinsic brain activity among MDD and MDD-A. In people who suffer from MDD, it has been previously shown that the left middle temporal gyrus is less engaged during emotional processing [61, 65, 66]. Furthermore, R-fMRI dynamic functional connectivity research has shown the ability to distinguish between postpartum depression, postpartum depression with anxiety symptoms, and healthy postpartum women based on the dynamic functional connectivity between the middle temporal gyrus and the subgenual anterior cingulate cortex [27]. Similarly, abnormalities in grey matter volume and fractional amplitude of low-frequency fluctuations were found in the left middle temporal gyrus of patients with anxious depression when comparing brain structure and function in patients with and without anxious depression [67]. Furthermore, the diagnosis and classification of generalized anxiety disorder and MDD may be aided by multilayered dynamic functional connectivity based on graph theory and gradient analysis, in particular functional connectivity gradients in middle temporal gyrus-subcortical regions [68]. Our findings are in accord with recent studies indicating that abnormal activity in the left middle temporal gyrus has been hypothesized to have a neurobiological foundation for MDD patients.

Our study revealed a negative correlation between anxiety levels in patients with MDD and abnormal coupling in the left medial superior frontal gyrus. The superior frontal gyrus, part of the default-mode network, is crucial for cognitive control and emotional regulation [69, 70]. Previous research has linked it to subjective stress perception [71, 72]. In addition, studies have shown that spontaneous brain activity in the superior frontal gyrus is correlated with anxiety levels in MDD, and that decreased activation in this region negatively impacts anxiety levels [60, 61, 73]. Bishop's neurocognitive model of anxiety [64] proposes that cognitive processes of anxiety display a selective bias towards threatening stimuli, which is modulated by the relative signal strength of top-down frontal control mechanisms. Another study indicates that abnormalities in neural circuits related to threat processing contribute to the emergence and progression of anxiety symptoms [74]. This dysregulation of cognitive neural circuitry, which is specifically associated with anxiety symptoms, can be recognized as a characteristic of MDD with anxiety symptoms. Our study presents evidence of the unique neurobiology of MDD with anxiety symptoms and suggests that abnormal coupling in the left medial superior frontal gyrus may serve as a marker for distinguishing MDD-A from MDD.

## 5. Conclusions

In conclusion, MDD and MDD-A patients have neurological abnormalities in frontal, parietal, occipital, and temporal brain activity, indicating poor affective and cognitive functioning. The coupling of the left medial superior frontal gyrus was inversely linked with HAMA scores, showing that anxiety levels in MDD are affected by insufficient involvement of frontal areas. Our research provides new insights into the neuropathology of MDD-A from a dynamic perspective. Our findings may have implications for the more precise identification of treatment targets for patients with MDD-A.

## 6. Limitations and Future Directions

First, we employed a cross-sectional approach, which is weak in examining causality. Secondly, our sample size is inadequate, which could explain our inability to detect more MDD-A-specific changes in brain regions. Future research could directly investigate how anxiety levels impact the function of the intrinsic brain in MDD. Thirdly, the level of anxiety in individuals with MDD was evaluated using HAMA, which is subject to more subjective biases. It is advisable that future assessments conduct an objective tool.

## Figures and Tables

**Table 1:** Demographic and clinical characteristics of MDD with anxiety, MDD without anxiety, and HC groups.

	MDD with anxiety (*n* = 45)	MDD without anxiety (*n* = 30)	HCs (*n* = 46)	*F*/*t*/*x*^2^	*p*
Age (years), mean ± SD	28.33 ± 6.657	29.07 ± 7.913	27.28 ± 6.065	0.666	0.516
Gender (male/female)	23/22	11/19	17/29	2.983	0.084
Educational level (years), mean ± SD	12.82 ± 3.4	13.73 ± 3.35	14.54 ± 2.335	3.686	0.028
HAMD score	29.8 ± 8.368	29.73 ± 5.458	—	0.001	0.969
HAMA score	24.09 ± 6.687	6.7 ± 4.276	—	2.881	0.004
Mean FD (mm)	0.0569 ± 0.0218	0.0582 ± 0.0219	0.056 ± 0.015	0.055	0.946

MDD: major depressive disorder; HCs: healthy controls; HAMD: Hamilton depression scale; HAMA: Hamilton anxiety scale; FD: framewise displacement.

**Table 2:** Brain regions with differences in R-fMRI index.

	Anatomical region	Peak MNI			Cluster size	*F*
		*x*	*y*	*z*		
Dynamic ALFF	Left calcarine sulcus	3	-93	3	26	10.6594
	Right calcarine sulcus	18	-57	3	18	10.3435
	Left postcentral gyrus	-51	-9	42	18	14.4252
	Left inferior parietal lobe	-42	-39	51	11	14.4616

Dynamic DC	Right lingual gyrus	6	-72	0	27	11.297
	Right calcarine sulcus	6	-63	18	58	14.9287

Dynamic GSCorr	Right middle occipital gyrus	36	-87	9	6	14.3714

Dynamic ReHo	Right middle occipital gyrus	42	-87	15	5	11.4952

ALFF: amplitude of low-frequency fluctuation; DC: degree centrality; GSCorr: global signal correlation; ReHo: regional homogeneity.

**Table 3:** Multiple comparisons of brain regions with differences in R-fMRI index.

rs-fMRI index	Brain region	(*I*)	(*J*)	Mean difference (*I*-*J*)	SD	*p*	95% confidence interval	
dynamic ALFF	Left calcarine sulcus	MDD with anxiety	MDD without anxiety	-0.0621	0.0783	0.429	-0.2172	0.0929
			HCs	-0.3317⁣^∗^	0.0696	<0.001	-0.4696	-0.1937
		MDD without anxiety	HCs	-0.2696⁣^∗^	0.0779	0.001	-0.4240	-0.1152
	Right calcarine sulcus	MDD with anxiety	MDD without anxiety	-0.0126	0.0729	0.863	-0.1571	0.1318
			HCs	-0.2879⁣^∗^	0.0649	<0.001	-0.4164	-0.1593
		MDD without anxiety	HCs	-0.2752⁣^∗^	0.0726	<0.001	-0.4191	-0.1313
	Left postcentral gyrus	MDD with anxiety	MDD without anxiety	-0.0331	0.0252	0.192	-0.0831	0.0169
			HCs	-0.1487⁣^∗^	0.0224	<0.001	-0.1932	-0.1042
		MDD without anxiety	HCs	-0.1156⁣^∗^	0.0251	<0.001	-0.1654	-0.0658
	Left inferior parietal lobe	MDD with anxiety	MDD without anxiety	-0.0246	0.0242	0.311	-0.0727	0.0233
			HCs	-0.1143⁣^∗^	0.0215	<0.001	-0.1571	-0.0716
		MDD without anxiety	HCs	-0.0896⁣^∗^	0.0241	<0.001	-0.1375	-0.0418

dynamic DC	Right lingual gyrus	MDD with anxiety	MDD without anxiety	-148.633	214.443	0.49	-573.29	276.02
			HCs	-899.487⁣^∗^	190.758	<0.001	-1277.24	-521.73
		MDD without anxiety	HCs	-750.854⁣^∗^	213.508	0.001	-1173.66	-328.05
	Right calcarine sulcus	MDD with anxiety	MDD without anxiety	-202.333	201.616	0.318	-601.59	196.92
			HCs	-912.464⁣^∗^	179.348	<0.001	-1267.62	-557.31
		MDD without anxiety	HCs	-710.130⁣^∗^	200.738	0.001	-1107.65	-312.62

dynamic GSCorr	Right middle occipital gyrus	MDD with anxiety	MDD without anxiety	-0.0184	0.0100	0.069	-0.0384	0.0014
			HCs	-0.0521⁣^∗^	0.0089	<0.001	-0.0698	-0.0343
		MDD without anxiety	HCs	-0.0336⁣^∗^	0.0100	0.001	-0.0535	-0.0137

dynamic ReHo	Right middle occipital gyrus	MDD with anxiety	MDD without anxiety	-0.0077	0.0032	0.981	-0.0065	0.0063
			HCs	-0.0130⁣^∗^	0.0029	<0.001	-0.0188	-0.0073
		MDD without anxiety	HCs	-0.0129⁣^∗^	0.0032	<0.001	-0.0194	-0.0065

MDD: major depressive disorder; HCs: healthy controls; SD: standard deviation; ALFF: amplitude of low-frequency fluctuation; DC: degree centrality; GSCorr: global signal correlation; ReHo: regional homogeneity. ⁣^∗^The mean difference is significant at the 0.05 level.

**Table 4:** Volume-wise concordance.

	MDD with anxiety	MDD without anxiety	HCs	*F*	*p*
Mean	0.5204 ± 0.0434	0.5248 ± 0.04853	0.5538 ± 0.04046	7.559	0.001
SD	0.0361 ± 0.01341	0.0362 ± 0.01284	0.0360 ± 0.0100	0.003	0.997

MDD: major depressive disorder; HCs: healthy controls; SD: standard deviation.

**Table 5:** Multiple comparisons of volume-wise concordance.

	(*I*)	(*J*)	Mean difference (*I*-*J*)	*p*	95% confidence interval	
Mean	MDD with anxiety	MDD without anxiety	-0.0043	0.673	-0.0247	0.0160
		HCs	-0.03336⁣^∗^	<0.001	-0.0514	-0.0152
	MDD without anxiety	HCs	-0.02899⁣^∗^	0.005	-0.0492	-0.0087

SD	MDD with anxiety	MDD without anxiety	-0.0001	0.964	-0.0057	0.0055
		HCs	0.0009	0.971	-0.0049	0.0051
	MDD without anxiety	HCs	0.0002	0.937	-0.005	0.0058

MDD: major depressive disorder; HCs: healthy controls; SD: standard deviation. ⁣^∗^The mean difference is significant at the 0.05 level.

**Table 6:** Brain regions with differences in voxel-wise concordance.

Anatomical region	Peak MNI			Cluster size	*F*
	*x*	*y*	*z*		
Left middle temporal gyrus	-60	-24	-6	8	15.3212
Left calcarine sulcus	-21	-69	12	5	12.349
Left medial superior frontal gyrus	-3	54	30	5	12.6044

**Table 7:** Multiple comparisons of voxel-wise concordance.

	(*I*)	(*J*)	Mean difference (*I*-*J*)	SD	*p*	95% confidence interval	
Left middle temporal gyrus	MDD with anxiety	MDD without anxiety	-0.0129	0.0225	0.567	-0.0574	0.0316
		HCs	-0.1287⁣^∗^	0.0200	<0.001	-0.1683	-0.089
	MDD without anxiety	HCs	-0.1157⁣^∗^	0.0224	<0.001	-0.1601	-0.0714

Left calcarine sulcus	MDD with anxiety	MDD without anxiety	0.0012	0.0224	0.955	-0.0431	0.0457
		HCs	-0.0997⁣^∗^	0.0199	<0.001	-0.1392	-0.0602
	MDD without anxiety	HCs	-0.1010⁣^∗^	0.0223	<0.001	-0.1452	-0.0567

left medial superior frontal gyrus	MDD with anxiety	MDD without anxiety	-0.0645⁣^∗^	0.0226	0.005	-0.1095	-0.0196
		HCs	-0.1216⁣^∗^	0.0201	<0.001	-0.1616	-0.0816
	MDD without anxiety	HCs	-0.0570⁣^∗^	0.022	0.013	-0.1018	-0.0123

MDD: major depressive disorder; HCs: healthy controls; SD: standard deviation.

**Table 8:** Correlations analysis of voxel-wise concordance and score of HAMD and HAMA.

	Left middle temporal gyrus	Left calcarine sulcus	Left medial superior frontal gyrus
HAMD score	-0.047	-0.03	0.095
HAMA score	0.047	0.087	-0.228⁣^∗^

HAMD: Hamilton Depression Scale; HAMA: Hamilton Anxiety Scale. ⁣^∗^The mean difference is significant at the 0.05 level.

## Data Availability

All data analysed during this study are included in this published article.
